# Supplementary material for: Protocols for Monitoring Harmful Algal Blooms for Sustainable Aquaculture and Coastal Fisheries in Chile
Source: Int J Environ Res Public Health. 2020 Oct 20;17(20):7642. doi: 10.3390/ijerph17207642 (PMC7589761; doi:10.3390/ijerph17207642)
Supplement: Supplementary file 1 [file ijerph-17-07642-s001.pdf]

Protocols for monitoring Harmful Algal Blooms for sustainable aquaculture and coastal fisheries in Chile (Supplement data)

Provided by Kyoko Yarimizu, et al.

Table S1. Phytoplankton Naming Dictionary: This dictionary was constructed from the species observed in Chilean coast water in the past combined with the IOC list. Each name was verified with the list provided by IFOP and online dictionaries, AlgaeBase (https://www.algaebase.org/) and WoRMS (http://www.marinespecies.org/). The list is subjected to be updated.

Phylum	Class	Order	Family	Genus	Species
Ochrophyta	Bacillariophyceae	Achnanthales	Achnanthaceae	Achnanthes	Achnanthes longipes
Bacillariophyta	Coscinodiscophyceae	Coscinodiscales	Heliopeltaceae	Actinoptychus	Actinoptychus spp.
Dinoflagellata	Dinophyceae	Gymnodiniales	Gymnodiniaceae	Akashiwo	Akashiwo sanguinea
Dinoflagellata	Dinophyceae	Gymnodiniales	Gymnodiniaceae	Amphidinium	Amphidinium spp.
Ochrophyta	Bacillariophyceae	Naviculales	Amphipleuraceae	Amphiprora	Amphiprora spp.
Bacillariophyta	Bacillariophyceae	Thalassiophysales	Catenulaceae	Amphora	Amphora spp.
Cyanobacteria	Cyanophyceae	Nostocales	Aphanizomenonaceae	Anabaenopsis	Anabaenopsis milleri
Cyanobacteria	Cyanophyceae	Oscillatoriales	Coleofasciculaceae	Anagnostidinema	Anagnostidinema amphibium
Cyanobacteria	Cyanophyceae	Oscillatoriales	Coleofasciculaceae	Anagnostidinema	Anagnostidinema lemmermannii
Cyanobacteria	Cyanophyceae	Oscillatoriales	Microcoleaceae	Annamia	Annamia toxica
Cyanobacteria	Cyanophyceae	Nostocales	Aphanizomenonaceae	Aphanizomenon	Aphanizomenon flos-aquae
Bacillariophyta	Bacillariophyceae	Rhaphoneidales	Asterionellopsidaceae	Asterionellopsis	Asterionellopsis glacialis
Bacillariophyta	Coscinodiscophyceae	Asterolamprales	Asterolampraceae	Asteromphalus	Asteromphalus heptactis
Bacillariophyta	Coscinodiscophyceae	Aulacoseirales	Aulacoseiraceae	Aulacoseira	Aulacoseira pseudogranulata
Ochrophyta	Pelagophyceae	Pelagomonadales	Pelagomonadaceae	Aureococcus	Aureococcus anophagefferens
Ochrophyta	Pelagophyceae	Sarcinochrysidales	Sarcinochrysidaceae	Aureoumbra	Aureoumbra lagunensis
Dinoflagellata	Dinophyceae	Incertae sedis	Amphidomataceae	Azadinium	Azadinium spinosum
Dinoflagellata	Dinophyceae	Incertae sedis	Amphidomataceae	Azadinium	Azadinium spp.
Haptophyta	Prymnesiophyceae	Coccosphaerales	Braarudosphaeraceae	Baarudosphera	Baarudosphera bigelowii
Bacillariophyta	Bacillariophyceae	Bacillariales	Bacillariaceae	Bacillaria	Bacillaria paxillifera
Bacillariophyta	Mediophyceae	Hemiaulales	Hemiaulaceae	Cerataulina	Cerataulina pelagica
Dinoflagellata	Dinophyceae	Gonyaulacales	Ceratiaceae	Ceratium	Ceratium balechi
Dinoflagellata	Dinophyceae	Gonyaulacales	Ceratiaceae	Ceratium	Ceratium furca
Dinoflagellata	Dinophyceae	Gonyaulacales	Ceratiaceae	Ceratium	Ceratium fusus

Phylum	Class	Order	Family	Genus	Species
Dinoflagellata	Dinophyceae	Gonyaulacales	Ceratiaceae	Ceratium	Ceratium spp.
Dinoflagellata	Dinophyceae	Gonyaulacales	Ceratiaceae	Ceratium	Ceratium tripos
Ochrophyta	Bacillariophyceae	Chaetocerotanae incertae sedis	Chaetocerotaceae	Chaetoceros	Chaetoceros spp.
Ochrophyta	Raphidophyceae	Chattonellales	Chattonellaceae	Chattonella	Chattonella antiqua
Ochrophyta	Raphidophyceae	Chattonellales	Chattonellaceae	Chattonella	Chattonella marina
Ochrophyta	Raphidophyceae	Chattonellales	Chattonellaceae	Chattonella	Chattonella ovata
Ochrophyta	Raphidophyceae	Chattonellales	Chattonellaceae	Chattonella	Chattonella subsalsa
Haptophyta	Coccolithophyceae	Prymnesiales	Chrysochromulinaceae	Chrysochromulina	Chrysochromulina leadbeateri
Haptophyta	Coccolithophyceae	Prymnesiales	Chrysochromulinaceae	Chrysochromulina	Chrysochromulina spp.
Cyanobacteria	Cyanophyceae	Nostocales	Aphanizomenonaceae	Chrysosporum	Chrysosporum bergii
Ciliophora					Ciliate
Ochrophyta	Bacillariophyceae	Climacospheniales	Climacospheniaceae	Climacosphenia	Climacosphenia moniligera
Ochrophyta	Bacillariophyceae	Climacospheniales	Climacospheniaceae	Climacosphenia	Climacosphenia spp.
Bacillariophyta	Bacillariophyceae	Cocconeidales	Cocconeidaceae	Cocconeis	Cocconeis spp.
Cyanobacteria	Cyanophyceae	Synechococcales	Coelosphaeriaceae	Coelosphaerium	Coelosphaerium kuetzingianum
Bacillariophyta	Coscinodiscophyceae	Corethrales	Corethraceae	Corethron	Corethron hystrix
Bacillariophyta	Coscinodiscophyceae	Corethrales	Corethraceae	Corethron	Corethron pennatum
Bacillariophyta	Coscinodiscophyceae	Coscinodiscales	Coscinodiscaceae	Coscinodiscus	Coscinodiscus spp.
Cyanobacteria	Cyanophyceae	Synechococcales	Synechococcaceae	Cyanobium	Cyanobium bacillare
Cyanobacteria	Cyanophyceae	Nostocales	Nostocaceae	Cylindrospermum	Cylindrospermum stagnale
Bacillariophyta	Bacillariophyceae	Bacillariales	Bacillariaceae	Cylindrotheca	Cylindrotheca closterium
Bacillariophyta	Coscinodiscophyceae	Rhizosoleniales	Rhizosoleniaceae	Dactyliosolen	Dactyliosolen blavyanus
Bacillariophyta	Coscinodiscophyceae	Rhizosoleniales	Rhizosoleniaceae	Dactyliosolen	Dactyliosolen fragilissimus
Ochrophyta	Bacillariophyceae	Thalassiosirales	Skeletonemaceae	Detonula	Detonula pumila
Ochrophyta	Bacillariophyceae				Diatom undefined (single cell around)
Ochrophyta	Bacillariophyceae				Diatom undefined (single cell pennel)
Ochrophyta	Dictyochophyceae	Dictyochales	Dictyochaceae	Dictyocha	Dictyocha speculum
Myzozoa					Dinoflagellate undefined
Myzozoa	Dinophyceae	Dinophysiales	Dinophysiaceae	Dinophysis	Dinophysis acuminata
Myzozoa	Dinophyceae	Dinophysiales	Dinophysiaceae	Dinophysis	Dinophysis caudata

Phylum	Class	Order	Family	Genus	Species
Myzozoa	Dinophyceae	Dinophysiales	Dinophysiaceae	Dinophysis	Dinophysis spp.
Bacillariophyta	Bacillariophyceae	Naviculales	Diploneidaceae	Diploneis	Diploneis spp.
Bacillariophyta	Mediophyceae	Lithodesmiales	Lithodesmiaceae	Ditylum	Ditylum brightwellii
Cyanobacteria	Cyanophyceae	Nostocales	Aphanizomenonaceae	Dolichospermum	Dolichospermum flosaquae
Cyanobacteria	Cyanophyceae	Nostocales	Aphanizomenonaceae	Dolichospermum	Dolichospermum lemmermannii
Cyanobacteria	Cyanophyceae	Nostocales	Aphanizomenonaceae	Dolichospermum	Dolichospermum macrosporum
Cyanobacteria	Cyanophyceae	Nostocales	Aphanizomenonaceae	Dolichospermum	Dolichospermum mendotae
Cyanobacteria	Cyanophyceae	Nostocales	Aphanizomenonaceae	Dolichospermum	Dolichospermum planctonicum
Cyanobacteria	Cyanophyceae	Nostocales	Aphanizomenonaceae	Dolichospermum	Dolichospermum sigmoideum
Cyanobacteria	Cyanophyceae	Nostocales	Aphanizomenonaceae	Dolichospermum	Dolichospermum spiroides
Cercozoa	Thecofilosea	Ebriida	Ebriidae	Ebria	Ebria tripartita
Bacillariophyta	Bacillariophyceae	Rhopalodiales	Rhopalodiaceae	Epithemia	Epithemia zebra
Bacillariophyta	Mediophyceae	Hemiaulales	Hemiaulaceae	Eucampia	Eucampia cornuta
Bacillariophyta	Mediophyceae	Hemiaulales	Hemiaulaceae	Eucampia	Eucampia spp.
Bacillariophyta	Mediophyceae	Hemiaulales	Hemiaulaceae	Eucampia	Eucampia zodiacus
Euglenophyta	Euglenophyceae				Euglenoids
Euglenozoa	Euglenophyceae	Eutreptiiida	Eutreptiidae	Eutreptiella	Eutreptiella gymnastica
Ciliophora	Oligotrichea	Choreotrichida	Ptychocylididae	Favella	Favella ehrenbergii
Ciliophora	Oligotrichea	Choreotrichida	Ptychocylididae	Favella	Favella spp.
Ochrophyta	Raphidophyceae	Chattonellales	Fibrocapsaceae	Fibrocapsa	Fibrocapsa japonica
					Flagellate undefined
Retaria					Foraminifera
Bacillariophyta	Bacillariophyceae	Fragilariales	Fragilariaceae	Fragilaria	Fragilaria crotonensis
Bacillariophyta	Bacillariophyceae	Fragilariales	Fragilariaceae	Fragilaria	Fragilaria spp.
Cyanobacteria	Cyanophyceae	Nostocales	Gloeotrichiaceae	Gloeotrichia	Gloeotrichia echinulata
Myzozoa	Dinophyceae	Gonyaulacales	Gonyaulacaceae	Gonyaulax	Gonyaulax spp.
Ochrophyta	Bacillariophyceae	Striatellales	Striatellaceae	Grammatophora	Grammatophora spp.
Bacillariophyta	Coscinodiscophyceae	Rhizosoleniales	Rhizosoleniaceae	Guinardia	Guinardia delicatula
Bacillariophyta	Coscinodiscophyceae	Rhizosoleniales	Rhizosoleniaceae	Guinardia	Guinardia spp.
Bacillariophyta	Coscinodiscophyceae	Rhizosoleniales	Rhizosoleniaceae	Guinardia	Guinardia striata
Miozoa	Dinophyceae	Gymnodiniales	Gymnodiniaceae	Gymnodinium	Gymnodinium spp.

Phylum	Class	Order	Family	Genus	Species
Miozoa	Dinophyceae	Gymnodiniales	Gyrodiniaceae	Gyrodinium	Gyrodinium dominans
Miozoa	Dinophyceae	Gymnodiniales	Gyrodiniaceae	Gyrodinium	Gyrodinium flavum
Miozoa	Dinophyceae	Gymnodiniales	Gyrodiniaceae	Gyrodinium	Gyrodinium spirale
Miozoa	Dinophyceae	Gymnodiniales	Gyrodiniaceae	Gyrodinium	Gyrodinium spp.
Bacillariophyta	Bacillariophyceae	Naviculales	Amphipleuraceae	Halamphora	Halamphora coffeaeformis
Miozoa	Dinophyceae	Peridiniales	Heterocapsaceae	Heterocapsa	Heterocapsa spp.
Ochrophyta	Raphidophyceae	Chattonellales	Chattonellaceae	Heterosigma	Heterosigma akashiwo
Cyanobacteria	Cyanophyceae	Oscillatoriales	Microcoleaceae	Kamptonema	Kamptonema formosum
Miozoa	Dinophyceae	Gymnodiniales	Kareniaceae	Karenia	Karenia spp.
Miozoa	Dinophyceae	Gymnodiniales	Kareniaceae	Karlodinium	Karlodinium spp.
Miozoa	Dinophyceae	Tovelliales	Tovelliaceae	Katodinium	Katodinium glaucum
Ciliophora	Oligotrichea	Oligotrichida	Tontoniidae	Laboea	Laboea strobila
Ochrophyta	Bacillariophyceae	Thalassiosirales	Lauderiaceae	Lauderia	Lauderia annulata
Miozoa	Dinophyceae	Gymnodiniales	Gymnodiniaceae	Lepidodinium	Lepidodinium chlorophorum
Bacillariophyta	Mediophyceae	Chaetocerotales	Leptocylindraceae	Leptocylindrus	Leptocylindrus danicus
Bacillariophyta	Mediophyceae	Chaetocerotales	Leptocylindraceae	Leptocylindrus	Leptocylindrus mediterraneus
Bacillariophyta	Mediophyceae	Chaetocerotales	Leptocylindraceae	Leptocylindrus	Leptocylindrus minimus
Bacillariophyta	Bacillariophyceae	Licmophorales	Licmophoraceae	Licmophora	Licmophora abbreviara
Cyanobacteria	Cyanophyceae	Oscillatoriales	Microcoleaceae	Limnospira	Limnospira fusiformis
Cyanobacteria	Cyanophyceae	Synechococcales	Pseudanabaenaceae	Limnothrix	Limnothrix redekei
Bacillariophyta	Bacillariophyceae	Thalassionematales	Thalassionemataceae	Lioloma	Lioloma pacificum
Cyanobacteria	Cyanophyceae	Oscillatoriales	Oscillatoriaceae	Lyngbya	Lyngbya majuscula
Bacillariophyta	Bacillariophyceae	Lyrellales	Lyrellaceae	Lyrella	Lyrella lyra
Bacillariophyta	Coscinodiscophyceae	Melosirales	Melosiraceae	Melosira	Melosira spp.
Ochrophyta	Xanthophyceae	Mischococcales	Pleurochloridaceae	Meringosphaera	Meringosphaera spp.
Ciliophora	Litostomatea	Cyclotrichiida	Mesodiniidae	Mesodinium	Mesodinium rubrum
Cyanobacteria	Cyanophyceae	Chroococcales	Microcystaceae	Microcystis	Microcystis aeruginosa
Cyanobacteria	Cyanophyceae	Chroococcales	Microcystaceae	Microcystis	Microcystis botrys
Cyanobacteria	Cyanophyceae	Chroococcales	Microcystaceae	Microcystis	Microcystis flosaquae
Cyanobacteria	Cyanophyceae	Chroococcales	Microcystaceae	Microcystis	Microcystis ichthyoblabe
Cyanobacteria	Cyanophyceae	Chroococcales	Microcystaceae	Microcystis	Microcystis panniformis
Cyanobacteria	Cyanophyceae	Chroococcales	Microcystaceae	Microcystis	Microcystis viridis
Cyanobacteria	Cyanophyceae	Chroococcales	Microcystaceae	Microcystis	Microcystis wesenbergii
Ciliophora	Litostomatea	Cyclotrichiida	Mesodiniidae	Myrionecta	Myrionecta rubra

Phylum	Class	Order	Family	Genus	Species
Bacillariophyta	Bacillariophyceae	Naviculales	Naviculaceae	Navicula	Navicula ammophila
Bacillariophyta	Bacillariophyceae	Naviculales	Naviculaceae	Navicula	Navicula spp.
Ochrophyta	Bacillariophyceae	Bacillariales	Bacillariaceae	Nitzschia	Nitzschia bizertensis
Ochrophyta	Bacillariophyceae	Bacillariales	Bacillariaceae	Nitzschia	Nitzschia longissima
Ochrophyta	Bacillariophyceae	Bacillariales	Bacillariaceae	Nitzschia	Nitzschia navis-varingica
Cyanobacteria	Cyanophyceae	Nostocales	Aphanizomenonaceae	Nodularia	Nodularia spumigena
Bacillariophyta	Mediophyceae	Eupodiscales	Odontellaceae	Odontella	Odontella longicruris
Bacillariophyta	Mediophyceae	Eupodiscales	Odontellaceae	Odontella	Odontella spp.
Chlorophyta	Ulvophyceae	Oltmannsiellopsidales	Oltmannsiellopsidaceae	Oltmannsiellopsis	Oltmannsiellopsis spp.
Miozoa	Dinophyceae	Dinophysales	Oxyphysaceae	Oxyphysis	Oxyphysis spp.
Miozoa	Dinophyceae	Peridiniales	Oxytoxaceae	Oxytoxum	Oxytoxum scolopax
Miozoa	Dinophyceae	Peridiniales	Oxytoxaceae	Oxytoxum	Oxytoxum spp.
Bacillariophyta	Coscinodiscophyceae	Paraliales	Paraliaceae	Paralia	Paralia sulcata
Haptophyta	Coccolithophyceae	Phaeocystales	Phaeocystaceae	Phaeocystis	Phaeocystis globosa
Haptophyta	Coccolithophyceae	Phaeocystales	Phaeocystaceae	Phaeocystis	Phaeocystis pouchetii
Miozoa	Dinophyceae	Dinophysales	Oxyphysaceae	Phalacroma	Phalacroma spp.
Cyanobacteria	Cyanophyceae	Oscillatoriales	Oscillatoriaceae	Phormidium	Phormidium uncinatum
Miozoa	Dinophyceae	Gonyaulacales	Pyrophacaceae	Pyrophacus	Pyrophacus steinii
Bacillariophyta	Bacillariophyceae	Naviculales	Pinnulariaceae	Pinnularia	Pinnularia spp.
Bacillariophyta	Bacillariophyceae	Naviculales	Plagiotropidaceae	Plagiotropis	Plagiotropis gaussii
Cyanobacteria	Cyanophyceae	Oscillatoriales	Microcoleaceae	Planktothrix	Planktothrix agardhii
Cyanobacteria	Cyanophyceae	Oscillatoriales	Prorocentrum+A137:E137	Planktothrix	Planktothrix rubescens
Ochrophyta	Bacillariophyceae	Naviculales	Pleurosigmataceae	Pleurosigma	Pleurosigma spp.
Miozoa	Dinophyceae	Gymnodiniales	Gymnodiniaceae	Polykrikos	Polykrikos spp.
Bacillariophyta	Coscinodiscophyceae	Rhizosoleniales	Probosciaceae	Proboscia	Proboscia alata
Miozoa	Dinophyceae	Prorocentrales	Prorocentraceae	Prorocentrum	Prorocentrum gracile
Miozoa	Dinophyceae	Prorocentrales	Prorocentraceae	Prorocentrum	Prorocentrum micans
Miozoa	Dinophyceae	Prorocentrales	Prorocentraceae	Prorocentrum	Prorocentrum spp.
Miozoa	Dinophyceae	Prorocentrales	Prorocentraceae	Prorocentrum	Prorocentrum triestinum
Dinophyceae	Dinophyceae	Gonyaulacales	Gonyaulacaceae	Protoceratium	Protoceratium spp.
Miozoa	Dinophyceae	Peridiniales	Protoperidiniaceae	Protoperidinium	Protoperidinium bipes
Miozoa	Dinophyceae	Peridiniales	Protoperidiniaceae	Protoperidinium	Protoperidinium conicum
Miozoa	Dinophyceae	Peridiniales	Protoperidiniaceae	Protoperidinium	Protoperidinium pellucidum
Miozoa	Dinophyceae	Peridiniales	Protoperidiniaceae	Protoperidinium	Protoperidinium spp.

Phylum	Class	Order	Family	Genus	Species
Miozoa	Dinophyceae	Peridiniales	Protoperidiniaceae	Protoperidinium	Protoperidinium steinii
Haptophyta	Coccolithophyceae	Prymnesiales	Prymnesiaceae	Prymnesium	Prymnesium calathiferum
Haptophyta	Coccolithophyceae	Prymnesiales	Prymnesiaceae	Prymnesium	Prymnesium faveolatum
Haptophyta	Coccolithophyceae	Prymnesiales	Prymnesiaceae	Prymnesium	Prymnesium parvum
Haptophyta	Coccolithophyceae	Prymnesiales	Prymnesiaceae	Prymnesium	Prymnesium polylepis
Haptophyta	Coccolithophyceae	Prymnesiales	Prymnesiaceae	Prymnesium	Prymnesium zebrinum
Ochrophyta	Dictyochophyceae	Florenciellales	Florenciellales incertae sedis	Pseudochattonella	Pseudochattonella farcimen
Ochrophyta	Dictyochophyceae	Florenciellales	Florenciellales incertae sedis	Pseudochattonella	Pseudochattonella verruculosa
Bacillariophyta	Bacillariophyceae	Bacillariales	Bacillariaceae	Pseudo-nitzschia	Pseudo-nitzschia spp.
Bacillariophyta	Bacillariophyceae	Bacillariales	Bacillariaceae	Pseudo-nitzschia	Pseudo-nitzschia abrensis
Bacillariophyta	Bacillariophyceae	Bacillariales	Bacillariaceae	Pseudo-nitzschia	Pseudo-nitzschia australis
Bacillariophyta	Bacillariophyceae	Bacillariales	Bacillariaceae	Pseudo-nitzschia	Pseudo-nitzschia batesiana
Bacillariophyta	Bacillariophyceae	Bacillariales	Bacillariaceae	Pseudo-nitzschia	Pseudo-nitzschia brasiliana
Bacillariophyta	Bacillariophyceae	Bacillariales	Bacillariaceae	Pseudo-nitzschia	Pseudo-nitzschia caciantha
Bacillariophyta	Bacillariophyceae	Bacillariales	Bacillariaceae	Pseudo-nitzschia	Pseudo-nitzschia calliantha
Bacillariophyta	Bacillariophyceae	Bacillariales	Bacillariaceae	Pseudo-nitzschia	Pseudo-nitzschia cuspidata
Bacillariophyta	Bacillariophyceae	Bacillariales	Bacillariaceae	Pseudo-nitzschia	Pseudo-nitzschia delicatissima
Bacillariophyta	Bacillariophyceae	Bacillariales	Bacillariaceae	Pseudo-nitzschia	Pseudo-nitzschia fraudulenta
Bacillariophyta	Bacillariophyceae	Bacillariales	Bacillariaceae	Pseudo-nitzschia	Pseudo-nitzschia fukuyoi
Bacillariophyta	Bacillariophyceae	Bacillariales	Bacillariaceae	Pseudo-nitzschia	Pseudo-nitzschia galaxiae
Bacillariophyta	Bacillariophyceae	Bacillariales	Bacillariaceae	Pseudo-nitzschia	Pseudo-nitzschia granii
Bacillariophyta	Bacillariophyceae	Bacillariales	Bacillariaceae	Pseudo-nitzschia	Pseudo-nitzschia hasleana
Bacillariophyta	Bacillariophyceae	Bacillariales	Bacillariaceae	Pseudo-nitzschia	Pseudo-nitzschia kodamae
Bacillariophyta	Bacillariophyceae	Bacillariales	Bacillariaceae	Pseudo-nitzschia	Pseudo-nitzschia lundholmiae
Bacillariophyta	Bacillariophyceae	Bacillariales	Bacillariaceae	Pseudo-nitzschia	Pseudo-nitzschia multiseries
Bacillariophyta	Bacillariophyceae	Bacillariales	Bacillariaceae	Pseudo-nitzschia	Pseudo-nitzschia multistriata
Bacillariophyta	Bacillariophyceae	Bacillariales	Bacillariaceae	Pseudo-nitzschia	Pseudo-nitzschia obtusa
Bacillariophyta	Bacillariophyceae	Bacillariales	Bacillariaceae	Pseudo-nitzschia	Pseudo-nitzschia plurisecta
Bacillariophyta	Bacillariophyceae	Bacillariales	Bacillariaceae	Pseudo-nitzschia	Pseudo-nitzschia pseudodelicatissima
Bacillariophyta	Bacillariophyceae	Bacillariales	Bacillariaceae	Pseudo-nitzschia	Pseudo-nitzschia pungens
Bacillariophyta	Bacillariophyceae	Bacillariales	Bacillariaceae	Pseudo-nitzschia	Pseudo-nitzschia seriata
Bacillariophyta	Bacillariophyceae	Bacillariales	Bacillariaceae	Pseudo-nitzschia	Pseudo-nitzschia simulans

Phylum	Class	Order	Family	Genus	Species
Bacillariophyta	Bacillariophyceae	Bacillariales	Bacillariaceae	Pseudo-nitzschia	Pseudo-nitzschia subfraudulenta
Bacillariophyta	Bacillariophyceae	Bacillariales	Bacillariaceae	Pseudo-nitzschia	Pseudo-nitzschia subpacifica
Bacillariophyta	Bacillariophyceae	Bacillariales	Bacillariaceae	Pseudo-nitzschia	Pseudo-nitzschia turgidula
Miozoa	Dinophyceae	Pyrocystales	Pyrocystaceae	Pyrocystis	Pyrocystis lunula
Cyanobacteria	Cyanophyceae	Nostocales	Aphanizomenonaceae	Raphidiopsis	Raphidiopsis curvata
Cyanobacteria	Cyanophyceae	Nostocales	Aphanizomenonaceae	Raphidiopsis	Raphidiopsis mediterranea
Cyanobacteria	Cyanophyceae	Nostocales	Aphanizomenonaceae	Raphidiopsis	Raphidiopsis raciborskii
Bacillariophyta	Bacillariophyceae	Rhabdonematales	Rhabdonemataceae	Rhabdonema	Rhabdonema spp.
Bacillariophyta	Coscinodiscophyceae	Rhizosoleniales	Rhizosoleniaceae	Rhizosolenia	Rhizosolenia imbricata
Bacillariophyta	Coscinodiscophyceae	Rhizosoleniales	Rhizosoleniaceae	Rhizosolenia	Rhizosolenia pungens
Bacillariophyta	Coscinodiscophyceae	Rhizosoleniales	Rhizosoleniaceae	Rhizosolenia	Rhizosolenia setigera
Bacillariophyta	Coscinodiscophyceae	Rhizosoleniales	Rhizosoleniaceae	Rhizosolenia	Rhizosolenia spp.
Bacillariophyta	Coscinodiscophyceae	Rhizosoleniales	Rhizosoleniaceae	Rhizosolenia	Rhizosolenia styliformis
Bacillariophyta	Bacillariophyceae	Cymbellales	Rhoicospheniaceae	Rhoicosphenia	Rhoicosphenia abbreviata
Miozoa	Dinophyceae	Thoracosphaerales	Thoracosphaeraceae	Scrippsiella	Scrippsiella spinifera
Miozoa	Dinophyceae	Thoracosphaerales	Thoracosphaeraceae	Scrippsiella	Scrippsiella spp.
Miozoa	Dinophyceae	Thoracosphaerales	Thoracosphaeraceae	Scrippsiella	Scrippsiella trochoidea
Bacillariophyta	Mediophyceae	Thalassiosirales	Skeletonemataceae	Skeletonema	Skeletonema spp.
Cyanobacteria	Cyanophyceae	Synechococcales	Coelosphaeriaceae	Snowella	Snowella lacustris
Bacillariophyta	Bacillariophyceae	Naviculales	Stauroneidaceae	Stauroneis	Stauroneis spp.
Bacillariophyta	Coscinodiscophyceae	Stephanopyxales	Stephanopyxidaceae	Stephanopyxis	Stephanopyxis nipponica
Ciliophora	Oligotrichea	Oligotrichida	Strombidiidae	Strombidium	Strombidium spp.
Bacillariophyta	Bacillariophyceae	Surirellales	Surirellaceae	Surirella	Surirella fastuosa
Bacillariophyta	Bacillariophyceae	Tabellariales	Tabellariaceae	Tabellaria	Tabellaria spp.
Bacillariophyta	Bacillariophyceae	Thalassionematales	Thalassionemataceae	Thalassionema	Thalassionema frauenfeldii
Bacillariophyta	Bacillariophyceae	Thalassionematales	Thalassionemataceae	Thalassionema	Thalassionema nitzschioides
Bacillariophyta	Mediophyceae	Thalassiosirales	Thalassiosiraceae	Thalassiosira	Thalassiosira eccentrica
Bacillariophyta	Mediophyceae	Thalassiosirales	Thalassiosiraceae	Thalassiosira	Thalassiosira rotula
Bacillariophyta	Mediophyceae	Thalassiosirales	Thalassiosiraceae	Thalassiosira	Thalassiosira spp.
Ciliophora	Spirotrichea	Tintinnida			Tintinnid
Miozoa	Dinophyceae	Gonyaulacales	Ceratiaceae	Tripos	Tripos lineatus
Myzozoa	Dinophyceae	Gymnodiniales	Gymnodiniaceae	Torodinium	Torodinium robustum
Cyanobacteria	Cyanophyceae	Nostocales	Nostocaceae	Trichormus	Trichormus variabilis

Phylum	Class	Order	Family	Genus	Species
Myzozoa	Dinophyceae	Gonyaulacales	Ceratiaceae	Tripos	Tripos furca
Myzozoa	Dinophyceae	Gonyaulacales	Ceratiaceae	Tripos	Tripos fusus
Myzozoa	Dinophyceae	Gonyaulacales	Ceratiaceae	Tripos	Tripos horridus
Ochrophyta	Dictyochophyceae	Dictyochales	Dictyochaceae	Vicicitus	Vicicitus globosus
